# Modernising the Repeat Prescription Service for ADHD Medications in a Community Mental Health Team (CMHT) in Cardiff, Wales

**DOI:** 10.1192/j.eurpsy.2026.11347

**Published:** 2026-07-31

**Authors:** E. Openshaw, J. Noblett

**Affiliations:** Psychiatry, Gabalfa Community Mental Health Team, Cardiff and Vale University Health Board, Cardiff, United Kingdom

## Abstract

**Introduction:**

ADHD is a neurodevelopmental disorder characterised by inattention, hyperactivity, and impulsivity. According to NICE, an estimated 3-4% of adults are diagnosed with ADHD in the UK. Gabalfa CMHT in Cardiff manages a large caseload, with ADHD making up a significant proportion. In 2025, an estimated 186 ADHD patients used the CMHT’s repeat prescription service. There is increasing demand for the provision of repeat prescription medication, as well as diagnosis, with almost 500 patients on the waiting list. The previous repeat prescription process for patients with ADHD was identified as inefficient. Requests were made either in person or via telephone, with patients verbally communicating their needs to administrative staff. The administrative staff (who also triage mental health queries and any emergencies) recorded the requests in a prescription book. Prescribers then accessed the book to review, print and sign prescriptions - a process that was often time-consuming.

**Objectives:**

To digitise the repeat prescription process within the CMHT, with the aim of improving patient accessibility and modernising the service.

**Methods:**

An NHS shared mailbox was introduced, which was accessible to the prescribers within the team, as well as administrative staff. The service was limited to core working hours to maintain clinical boundaries and minimise inappropriate or out-of-hours requests. Patients were informed of the change via SMS or email, including guidance on how to use the new system. Anonymous patient feedback was gathered via a survey form, sent by SMS.

**Results:**

Within 6 months of the email inbox being piloted, many patients had started to use the email inbox service. Twelve patients responded to the initial survey. 83% of these patients were ‘very satisfied’ or ‘satisfied’ with the new email service. 64% found this service more convenient. 67% of patients would prefer to continue to use the email service, rather than the previous method and 25% had no preference. In addition, staff feedback noted a significant decrease in the number of calls received relating to ADHD medication, thus reducing workload.

**Conclusions:**

The initial patient feedback demonstrates the positive impact of using digital tools in repeat prescription services. The key benefits are improved patient accessibility, reduced administrative burden on staff and increased efficiency. However, there is an ongoing digital divide, with some patients not using the service at all. The next steps would be to gain further feedback, to ensure a more representative patient opinion has been collected, over a longer period of time. With continued improvements to the new service, the CMHT could look at expanding to a wider patient group, not just those with ADHD.

**Disclosure of Interest:**

None Declared

